# [Retracted] Propofol exhibits a tumor‑suppressive effect and regulates cell viability, migration and invasion in bladder carcinoma by targeting the microRNA‑10b/HOXD10 signaling pathway 

**DOI:** 10.3892/ol.2024.14779

**Published:** 2024-10-29

**Authors:** Zongcai Qi, Lei Yuan, Nenghong Sun

Oncol Lett 18: 6228–6236, 2019; DOI: 10.3892/ol.2019.10968

Following the publication of this paper, it was drawn to the Editor's attention by a concerned reader that certain of the Matrigel invasion assay data shown in Fig. 1C and 4D on p. 6230 and p. 6233 respectively exhibited a number of unexpected repeating patterns of cellular data within the affected data panels themselves, which could not be easily accounted for by the coincidental arrangement of cells. The authors responded to the reader to say that the anomalies were due to inadequate processing of the images; however, after having considered this matter, the Editor of *Oncology Letters* has decided that this paper should be retracted from the Journal on the grounds of a lack of confidence in the presented data. The authors were asked for an explanation to account for these concerns, but the Editorial Office did not receive a satisfactory reply. The Editor apologizes to the readership for any inconvenience caused.

